# Cooperative activity of DNA methyltransferases for maintenance of symmetrical and non-symmetrical cytosine methylation in *Arabidopsis thaliana*

**DOI:** 10.1111/j.1365-313X.2008.03640.x

**Published:** 2008-08-26

**Authors:** Anuradha Singh, Elena Zubko, Peter Meyer

**Affiliations:** Centre for Plant Sciences, University of LeedsLeeds LS2 9JT, UK

**Keywords:** DNA methylation, MET1, DRM2, CMT3, DCM-like methylation

## Abstract

Maintenance of cytosine methylation in plants is controlled by three DNA methyltransferases. MET1 maintains CG methylation, and DRM1/2 and CMT3 act redundantly to enforce non-CG methylation. *RPS*, a repetitive hypermethylated DNA fragment from *Petunia hybrida*, attracts DNA methylation when transferred into *Petunia* or other species. In *Arabidopsis thaliana*, which does not contain any *RPS* homologues, *RPS* transgenes are efficiently methylated in all sequence contexts. To test which DNA methylation pathways regulate *RPS* methylation, we examined maintenance of *RPS* methylation in various mutant backgrounds. Surprisingly, CG methylation was lost in a *drm1/2/cmt3* mutant, and non-CG methylation was almost completely eliminated in a *met1* mutant. An unusual cooperative activity of all three DNA methyltransferases is therefore required for maintenance of both CG and non-CG methylation in *RPS*. Other unusual features of *RPS* methylation are the independence of its non-CG methylation from the RNA-directed DNA methylation (RdDM) pathway and the exceptional maintenance of methylation at a CC^m^TGG site in some epigenetic mutants. This is indicative of activity of a methylation system in plants that may have evolved from the DCM methylation system that controls CC^m^WGG methylation in bacteria. Our data suggest that strict separation of CG and non-CG methylation pathways does not apply to all target regions, and that caution is required in generalizing methylation data obtained for individual genomic regions.

## Introduction

DNA methylation has evolved from an immune function in bacteria to a regulator of gene expression and genome structure in higher eukaryotes. In bacteria, methylation targets are determined on the basis of their DNA sequence, as type II DNA methyltransferases methylate short recognition sequences, providing protection against methylation-sensitive endonucleases that target the same sequence (Wilson, 1988). In higher eukaryotes, DNA methylation systems have to fulfil new functions that are not compatible with the universal distribution of DNA methylation marks across the genome. For example, inactivation of parasitic sequences or compartmentalization of heterochromatic regions require selective establishment and clonal inheritance of DNA methylation patterns based on *de novo* and maintenance methylation systems. The evolution and adaptation of prokaryotic DNA methylation systems was probably a requirement for higher eukaryotes to manage their large genomes (Bestor, 1990), and a comparison of the features of DNA methylation systems present in higher eukaryotes may help us to understand this evolutionary process.

Plants show three cytosine methylation types, CG, CNG and CNN methylation, which are regulated by three DNA methylation functions. DNMT1-like METHYLTRANSFERASE 1 (MET1) controls CG methylation (Finnegan and Dennis, 1993; Saze *et al.*, 2003). CHROMOMETHYLASE 3 (CMT3) is the main enzyme controlling methylation at CNG sites (Lindroth *et al.*, 2001) but also affects CNN methylation (Bartee *et al.*, 2001). Two members of the DOMAINS REARRANGED METHYLTRANSFERASE family, DRM1 and DRM2, are also responsible for CNN methylation (Cao and Jacobsen, 2002a), thus CNG and CNN methylation are therefore controlled redundantly by CMT3, DRM1 and DRM2 (Chan *et al.*, 2005). While the symmetry of CG methylation targets provides a means for maintenance after replication, non-symmetrical methylation (NSM) patterns can only be maintained via continuous *de novo* methylation, which requires defined helper functions. NSM patterns are maintained by three partially overlapping pathways that can be distinguished by their effects on individual target regions.

NSM of endogenous repeats at the flowering time locus FWA and at the repeat element MEA-ISR requires a 24 nt siRNA whose production is controlled by the two largest subunits of RNA-DEPENDENT POLYMERASE IV (NRPD1a and NRPD1b), RNA-DEPENDENT RNA POLYMERASE 2 (RDR2), DICER-LIKE 3 (DCL3) and ARGONAUTE4 (AGO4). It has been proposed that, within a nucleolar processing centre, NRPD1a-generated RNA is copied by RDR2 into dsRNA, which DCL3 cleaves into 24 nt siRNAs that assemble with an AGO4/NRPD1b-containing silencing complex (Li *et al.*, 2006; Pontes *et al.*, 2006). This complex guides DRM2 (the more highly expressed member of the DRM family; Cao and Jacobsen, 2002a) to its target regions, where it induces NSM (Chan *et al.*, 2004).

NSM at the SINE element *AtSN1* uses a combination of the RNAi pathway and a second pathway in which CMT3 is guided to the target region by histone H3 lysine 9 dimethylation (H3K9me2), which is established by the suppressor of variegation-enhancer of zeste-trithorax (SET) domain protein SUVH4/KRYPTONITE (KYP) (Jackson *et al.*, 2002). An essential regulator both for the RNAi pathway and the KYP-dependent pathway is the putative SNF2 chromatin remodelling protein DRD1 (Kanno *et al.*, 2004). DRD1 works together with the 24 nt siRNA pathway in the establishment of DNA methylation, with DRM2 and CMT3 maintaining DNA methylation (Chan *et al.*, 2006).

NSM at the pericentromeric retrotransposon *Ta3* does not require the siRNA pathway and is also independent of DRD1. Instead, *Ta3* methylation is regulated by CMT3- and KYP-based histone H3K9me2 methylation in an alternative DRD1-independent pathway (Chan *et al.*, 2006).

We have previously described the *RPS* sequence element from *Petunia hybrida*, which acts as a hot spot for *de novo* DNA methylation when transferred into the Arabidopsis genome (Muller *et al.*, 2002). There is no indication that the *RPS* element is transcribed, and it has been suggested that *RPS* attracts DNA methylation via its palindromic structures (Muller *et al.*, 2002). To test the influence of epigenetic regulators on *RPS*-specific methylation patterns, we crossed a methylated *RPS* transgene into various Arabidopsis mutant backgrounds. Surprisingly, we found that the three methyltransferases MET1, CMT3 and DRM1/2 are all required cooperatively for *RPS* methylation both at CG and non-CG sites.

## Results

**Figure 1:**
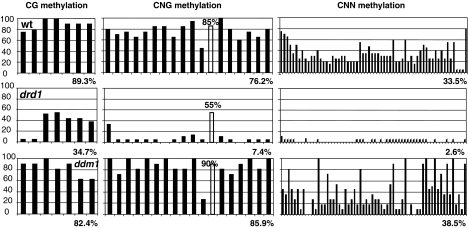
*RPS* methylation in putative chromatin remodelling mutants Methylation is reduced in the *drd1* mutant but unaffected in *ddm1*. Bars indicate methylation rates for individual C residues within the analysed *RPS* region, separated into three sequence-context groups. Overall methylation frequencies are listed below each graph. Open bars indicate the methylation frequency for the second C at a CCTGG site, which is only moderately reduced in the *drd1* mutant.

The transgenic Arabidopsis line RA5 contains a single copy of the *RPS* transgene, which is heavily methylated (Muller *et al.*, 2002). We used this line for crosses with epigenetic pathway mutants to test their effects on maintenance of RPS methylation. RPS methylation was first examined in the putative chromatin remodelling mutants *drd1* and *ddm1* (Figure 1). DDM1 had no influence on the methylation of cytosines in any context but methylation levels were significantly affected in *drd1*, especially at non-CG targets, as CNG and CNN methylation levels dropped to below 10% of the wild-type levels for these targets. CG-specific methylation was also reduced in *drd1*, especially at two CG sites in the 5′ region. In contrast to the significant hypomethylation of CNG sites, methylation at the second C residue within a CCTGG sequence was only moderately reduced in the *drd1* mutant.

**Figure 2:**
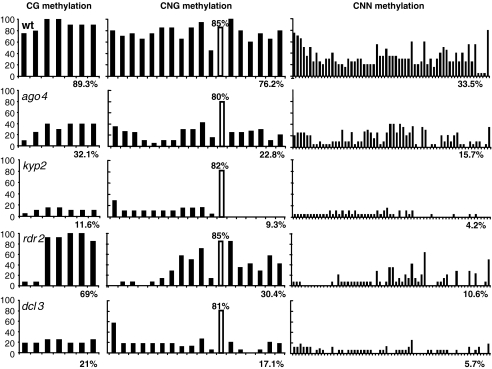
*RPS* methylation in mutants of the RNA-directed DNA methylation pathway All lines show some hypermethylation at CG and non-CG targets, except for the majority of CG sites in *rdr2*, for which the methylation state is maintained. In contrast to other CNG sites, CC^m^TGG methylation is not altered in any of the four mutants.

As a next step, we compared *RPS* methylation patterns in lines carrying mutations of four genes required for NSM mediated by the RNAi pathway or the KYP-dependent pathway (Figure 2). In all four lines, both CNG and CNN methylation levels were reduced, with *kyp2* and *dcl3* having the strongest impact. The only exception was again methylation at the CC^m^TGG site, which was not significantly altered in any of the four mutants. Surprisingly, CG methylation was also reduced in *kyp2*, *dcl3* and *ago4.* In *rdr2*, most CG targets remained hypermethylated, except for the two cytosines in the 5′ region that were also affected in *drd1*.

**Figure 3:**
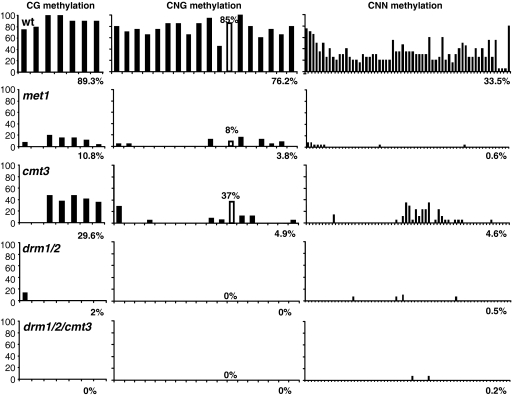
*RPS* methylation in DNA methyltransferase mutants DNA methylation is significantly reduced in all lines. Non-CG methylation is almost eliminated in *met1*, but a low level of CG methylation is maintained. No CG methylation and only traces of non-CG methylation are retained in the *drm1/2/cmt3* mutant.

*RPS* methylation was then tested in the three DNA methyltransferase mutants (Figure 3). Surprisingly, DNA methylation was significantly reduced in all lines irrespective of the sequence context. In *met1*, hypomethylation affected CG sites, and non-CG methylation was also almost eliminated. The mildest effects were detectable in *cmt3*, in which C residues in all sequence contexts were hypomethylated, but this effect was less pronounced at the CC^m^TGG site, at some central CNN sites and at CG sites in the 3′ region. The most significant hypomethylation effect was observed in the *drm1/2*mutant double mutant and a *drm1/2/cmt3* triple mutant, in which methylation was almost completely eliminated, with only some traces of CNN methylation. Maintenance of RPS-specific CG methylation can therefore not be guaranteed by MET1 alone but requires both DRM1/2 and CMT3. Equally, DRM1/2 and CMT3 are necessary but not sufficient for maintenance of CNG and CNN methylation, which also requires MET1. Maintenance of methylation at the CC^m^TGG site also required DRM1/2, MET1, and, to a lesser extent, CMT3.

**Figure 4:**
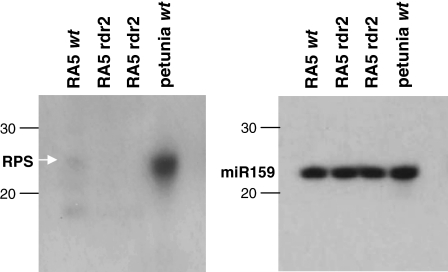
Analysis of *RPS*-specific small RNAs Small RNA fractions were prepared from RA5, from two *rdr2* lines with the RA5 transgene, and from wild-type petunia. Hybridization to an *RPS* probe detected a strong signal at approximately 24 nt in the petunia sample and a very weak corresponding signal in RA5; this signal is absent in the *rdr2* samples. Hybridization with an miR159 probe was used to determine equal loading of the RNA samples.

**Figure 5:**
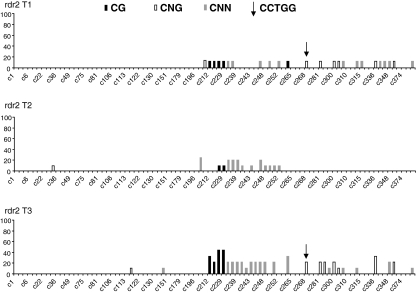
*De novo* methylation of *RPS* does not depend on RDR2 DNA methylation pattern of the *RPS* region in three transgenic *rdr2* mutant lines. In all transformants, a low level of *de novo* cytosine methylation was detectable in all sequence contexts.

Although some hypomethylation was detectable in *rdr2*, *kyp2*, *dcl3* and *ago4*, neither CNG nor CNN methylation were eliminated. This result was in accordance with the assumption that, while the RNAi pathway may augment *RPS* methylation, it is not essential for its maintenance. The analysis of *RPS*-specific small RNAs (Figure 4) also supports this model. *Petunia*, which contains a large pool of methylated *RPS* copies and *RPS* homologues, shows a strong signal for *RPS*-specific small RNAs. A very faint signal of similar size was also detectable in the RA5 line, which has a single methylated *RPS* copy, but the small RNA is no longer detectable in the *rdr2* line, which still shows a significant level of *RPS* methylation (Figure 2). The small RNA in RA5 may therefore reflect an enhancement of *RPS* methylation via the RNAi pathway, but basic levels of *RPS* methylation can be maintained without the RNAi pathway. To test whether this independence also applies to initiation of *RPS*-specific methylation, we transformed an *rdr2* mutant with an *RPS* construct and analysed methylation patterns in three independent transformants (Figure 5). All three lines showed a low but significant methylation level for the *RPS* transgene, which suggests that *RPS* methylation is not only maintained but can also be initiated in the absence of the RNAi pathway.

**Figure 6:**
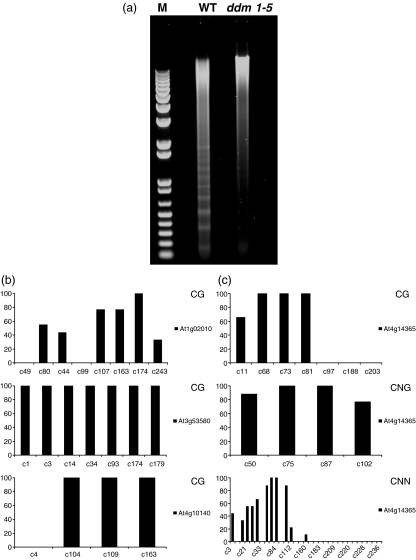
Methylation patterns of cloned genomic regions in *ddm*1 (a) Digestion of genomic DNA from wild-type and *ddm1* using *Gla*I, which requires cytosine methylation. Only wild-type DNA shows a pattern of *Gla*I restriction fragments indicative of methylated repetitive DNA. These regions are not digested in *ddm1*, as DDM1 is required for their methylation. (b) Methylation of CG sites in three cloned regions in *ddm1* genomic DNA. CG methylation is conserved in *ddm1* for all three clones. Bars show the methylation levels of C residues labeled according to their position on the sequenced fragments. (c) Methylation of CG, CNG and CNN sites in a cloned fragment near At4g14365 is conserved in *ddm1*.

Apart from the joint requirement for various methyltransferases and the independence of non-CG methylation from the small RNA pathway, another unusual feature of *RPS* was that its methylation was dependent on MET1 but not influenced by DDM1, as these two functions jointly regulate CG methylation for a number of endogenes and transgenic loci. To test whether the plant genome contains other regions with methylation patterns that are independent of DDM1, we cloned genomic DNA of a *ddm1* mutant after digestion with *Gla*I, which requires methylated cytosines for restriction. *Gla*I cleaves fully methylated CGCG sites, ACGC and GCGC sites which contain at least three methylated C residues, and GCGT sites with at least two methylated residues (Tarasova *et al.*, 2008). When digested with *Gla*I, *ddm1* genomic DNA no longer contains the characteristic bands that are indicative of methylated repetitive regions in the wild-type (Figure 6a). A faint background level suggested that a small fraction of the *ddm1* DNA was digested by *Gla*I. After cloning this fraction, we sequenced nine regions and used the highly integrated single-base resolution maps at http://neomorph.salk.edu/epigenome.html (Lister *et al.*, 2008) to examine their methylation profiles in wild-type and DNA methyltransferase mutants. Eight of the nine cloned regions represented genes with CG methylation regions located in the central and/or 3′ coding region. One clone comprised a methylated repeat region next to the 3′ UTR of At4g14365, which contained CG and non-CG methylation targets. In all cloned regions, CG methylation is abolished in *met1* and retained in the *drm1/2/cmt3* triple mutant. The non-CG methylation pattern in the region near the At4g14365 gene is lost in the *drm1/2/cmt3* triple mutant and retained in *met1* (Table 1). We selected regions from three clones with CG methylation (Figure 6b) and from the only clone with CG and non-CG methylation (Figure 6c) for bisulfate analysis of *ddm1* DNA, which confirmed that all clones maintained their methylation pattern in *ddm1*. Like *RPS* methylation, methylation in some euchromatic regions is therefore independent of DDM1. In contrast to *RPS*, however, CG and non-CG patterns in these regions are separately controlled by MET1 and DRM1/2/CMT3 activity.

**Table 1:** Clones isolated after *Gla*I digestion of *ddm1* genomic DNA

	Methylation in			
Gene	Wild-type	*met1*	*drm1/2/cmt3*	Encoded protein
At1g02010	CG		CG	SEC1A; protein transporter member of KEULE gene family
At3g01370	CG		CG	Unknown protein
At3g50040	CG		CG	Unknown protein
At3g53580	CG		CG	Unknown protein; similar to diaminopimelate epimerase, putative, expressed
At4g10140	CG		CG	Unknown protein
At4g14365	CG CNG CNN	CNG CNN	CG	Unknown protein; similar to zinc finger (C3HC4-type RING finger) family protein/ankyrin repeat family protein
At4g32910	CG		CG	Unknown protein; similar to Os01g0746200 (*Oryza sativa*, japonica cultivar group) (GB: NP_001044228.1)
At5g16780	CG		CG	Unknown protein; encodes a protein belonging to the SART-1 family
At5g22130	CG		CG	Unknown protein; member of glycosyltransferase family 50

## Discussion

The *RPS* element was selected in a screen for *Petunia* DNA elements that destabilize the expression of an adjacent marker gene (tenLohuis *et al.*, 1995). *RPS* belongs to a group of middle repetitive, dispersed and hypermethylated homologues. Repetitiveness, however, is not a prerequisite for hypermethylation, as *RPS* transgenes are efficient methylation targets in Arabidopsis, which lacks any significant *RPS* homology. As attempts to identify *RPS* transcripts had been unsuccessful, it had been proposed that RPS hypermethylation was independent of the RdDM pathway (Muller *et al.*, 2002). Our analysis of *RPS* methylation patterns in plants bearing mutations in chromatin-remodelling enzymes, RdDM pathway functions and DNA methyltransferases supports this model, and identifies some unusual requirements for maintenance of *RPS* methylation.

### RPS methylation is independent of DDM1

One surprising observation was that *RPS*-specific DNA methylation was unaltered in the *ddm1* mutant. DDM1 has similarities to members of the SWI/SNF family of adenosine triphosphate-dependent chromatin-remodelling proteins, suggesting an indirect role in DNA methylation, control of methylation and transcriptional inactivation of transposons (Miura *et al.*, 2001), heterochromatic repeats (Steimer *et al.*, 2000) and transgenes (Mittelsten Scheid *et al.*, 1998). In *ddm1* mutants, repetitive sequences are quickly demethylated, while hypomethylation of many low-copy regions occurs progressively (Jeddeloh *et al.*, 1998). In contrast, *RPS*-specific methylation is fully maintained in *ddm1*.

Hypermethylation of *RPS* in a *ddm1* background is surprising in view of the very strong hypomethylation of *RPS* in the *met1* mutant, as DDM1 and MET1 usually show close cooperativity. Efficient maintenance of RNA-directed DNA methylation requires the activity of DDM1 and MET1 (Aufsatz *et al.*, 2002), which are also essential for silencing of elements that are potentially independent of the RDM pathway and for which no small RNAs have been found (Rangwala and Richards, 2007). As far as we are aware, the only example of a locus that is differently affected by DDM1 and MET1 is *Sadhu6-1*, a non-autonomous retroposon that is reactivated in *met1* but not in *ddm1*; CG methylation levels for *Sadhu6-1* are reduced from 95% to 62% in *met1* but only to 83% in *ddm1* (Rangwala and Richards, 2007). For *RPS*, we see a similar but even more drastic discrepancy, with CG methylation levels decreasing from 89% to 11% in *met1* but only to 82% in *ddm1.*

Our search for other genomic regions with CG methylation patterns that remained unaltered in a *ddm1* mutant background identified several genes that all contained methylated CG blocks in the centre or 3′ half of their coding regions. As for *RPS*, CG methylation of all these genes was eliminated in *met1* (Lister *et al.*, 2008) but maintained in *ddm1*. DDM1 is therefore essential for DNA methylation of certain but not all genomic regions. It is tempting to speculate that repetitive or heterochromatic regions are prime targets for DDM1, while unique or euchromatic regions are methylated independently of DDM1. Repetitiveness may, however, not be sufficient for a region to come under DDM1 control, as we found a block of methylated CG and non-CG targets in a repetitive region near the 3′ UTR of At4g14365 that are also independent of DDM1.

### RdDM pathway functions enhance RPS methylation but are not essential for maintenance or initiation of RPS methylation

The general reduction of DNA methylation levels in *drd1*, *kyp2*, *dcl3*, *ago4* and *rdr2* suggests that RdDM pathways contribute to the maintenance of *RPS* methylation. However, this effect is not specific for non-CG methylation targets, and this is most obvious in *kyp2* and *dcl3* backgrounds for which CG methylation levels fall from 89% to 12% and 21%, respectively. This contrasts with reports regarding the conservation of CG methylation in *dcl3* at *MEA-ISR*, *AtSN1* and *IR-71* (Henderson *et al.*, 2006). For *kyp*, a moderate reduction of CG methylation from 16% to 6% has been reported for *Superman* (*SUP*), but CG methylation at *FWA*, *TSI*, *TA3* and at a 180-bp centromeric repeat remains unchanged (Jackson *et al.*, 2002).

Although *drd1*, *kyp2*, *dcl3*, *ago4* and *rdr2* all show a hypomethylation effect, none of the mutants inhibits *RPS* methylation completely, and a similar basic level of methylation is also observed in *RPS* transgenes after transfer into *rdr2.* The basic methylation level in all five mutants, the lack of an *RPS*-specific siRNA in the *rdr2* background and the failure to detect *RPS*-specific transcripts suggest that the initial *RPS* methylation level is established independently of RdRM pathways. The enhanced methylation levels and the presence of an *RPS*-specific siRNA in RA5 suggest that basal methylation levels are amplified via the RdRM pathway. It has been suggested that siRNA production requires NRPD1a to transcribe either a methylated target region (Herr *et al.*, 2005) or locus-specific nascent transcripts (Pontes *et al.*, 2006) or dsRNA (Vaucheret, 2005). Our data support a signal function for methylated RPS DNA in the initiation of siRNA production.

### The separation between CG and non-CG methylation pathways is lost in RPS

Another surprising feature of *RPS* methylation is the influence of the various DNA methyltransferases on methylation of cytosines outside their usual target sequence context. These characteristics are not shared by the cloned DDM1-independent methylation targets, for which we see a clear separation between MET1-controlled CG methylation and DRM1/2/CMT3-controlled non-CG methylation. MET1 was therefore expected to regulate *RPS*-specific CG methylation only, which did actually drop from 89 to 11% in *met1*. However, CNG methylation was also reduced from 76% to 3.8% and CNN methylation decreased from 33% to 0.6%. There are a few reports indicating that MET1 is important for the maintenance of CNG methylation at other loci (Rangwala and Richards, 2007), but these effects are relatively modest compared to the very drastic reduction of *RPS*-specific non-CG methylation in *met1*. Equally surprising, *RPS*-specific hypomethylation in the *drm1 drm2* background was not limited to non-CG targets but also included CG methylation, which decreased from 89 to 2% in *drm1/2* and was completely eliminated in the *drm1/2/cmt3* triple mutant. The presence of MET1 is therefore not sufficient to maintain CG methylation of *RPS*. This contrasts with reports on *FWA*, *MEA-IR* and *SUP*, for which CG methylation remains unaltered in the *drm1/2/cmt3* triple mutant, reflecting the primary importance of MET1 for CG methylation (Cao and Jacobsen, 2002b). In line with our observations for RPS, the *drm2* single mutation has been shown to cause a moderate reduction in both CG and CNG methylation at 5S rDNA (Mathieu *et al.*, 2007).

The unusual influence of DRM1/2, CMT3, DCL3 and KYP on the maintenance of CG methylation, and the participation of MET1 in maintaining CNG and CNN methylation, make *RPS* a very unusual methylation target. Our results suggest that maintenance of *RPS* methylation requires mutual enforcement of symmetrical and non-symmetrical DNA methylation systems, and that all methylation patterns are significantly reduced or lost if either of the two systems is compromised. This may reflect a cooperative effect whereby MET1, DRM1/2 and CMT3 only gain access to *RPS* jointly, or it may be the result of methylation-sensitive auxiliary factors that guide methyltransferases to *RPS*. The latter model implies that, for example, CG methylation is required for binding of DRM1/2 and CMT3 guiding factors, and non-CG methylation enables binding of the MET1 guiding factors. Loss of CG methylation would then compromise maintenance of non-CG methylation and *vice versa*.

### A DCM-like methylation site is independent of RDM functions

Although RPS is so far the only target requiring the cooperative activity of MET1, CMT3 and DRM1/2, its special regulation indicates that sequence- or locus-specific factors should be taken into account to understand the composition of DNA methylation patterns. This conclusion is also supported by the observation that CG methylation patterns at certain loci are controlled by DDM1, while CG targets at other loci are independent of DDM1. In addition, some of our results highlight how careful we need to be in interpreting methylation data for individual sites as indicators for a locus. Among the seven CG sites in the analysed *RPS* region, for example, we detected at two sites a 90% reduction of DNA methylation in *rdr2*, but methylation at the other five sites does not change at all. The most significant exception, however, is the conservation of CNG methylation at a CC^m^TGG site in RDM mutant backgrounds. This implies that CC^m^TGG methylation at this site is independent of a small RNA pathway. DRD1 may have a moderate influence on maintenance of CC^m^TGG methylation, probably by facilitating access to the region for regulatory proteins or methyltransferases.

The presence of a CCWGG methylation system in mammals illustrates the transmission of DCM-like methylation systems into eukaryotes. Initially, CC^m^WGG methylation was interpreted as maintenance of CNG methylation when it was detected in CC^m^WGG-methylated plasmid DNA that had been transferred into the genome of mouse cell lines (Clark *et al.*, 1995). The discovery of CC^m^WGG methylation in retroviral DNA (Lorincz *et al.*, 2000) and in endogenous promoter regions (Malone *et al.*, 2001), however, argues in favour of a *de novo* CC^m^WGG methylation activity in mammals. This is also the most likely explanation for our results. Due to the absence of CC^m^WGG methylation in *Agrobacterium tumefaciens* (Gomez-Eichelmann *et al.*, 1991), the transferred T-DNA is unmethylated when transferred into the plant genome, and CC^m^TGG methylation is established *de novo* in the transformed plant. CC^m^TGG methylation is significantly reduced or lost in all DNA methyltransferase mutants, which implies that MET1, DRM1/2 and CMT3 either help to recruit an unknown CCTGG methyltransferase to the *RPS* region, or that the CCTGG site is efficiently labelled as a target for methylation, mediated by joint activity of the three methyltransferases.

Our date demonstrate that, at least for certain loci, DNA methylation patterns cannot exclusively be interpreted as the result of a specific DNA methylation function or pathway. To fully understand the dynamics of DNA maintenance, it will be important to consider target-specific characteristics that influence the accessibility and cooperativity of methyltransferases or their auxiliary factors. This may also contribute to a better understanding of the high levels of methylation polymorphism (Vaughn *et al.*, 2007) and locus-specific methylation variation (Fischer *et al.*, 2008).

## Experimental procedures

### Plant material

All plants were grown under 8 h short-day conditions at 22°C. *Arabidopsis thaliana* mutants used in this study and their ecotypes are described in Appendix S1. The Arabidopsis line RA5 containing a single copy of a p35S *GUS/RPS* transgene (Muller *et al.*, 2002) was used for crosses with the various mutants. Progeny plants were selfed, and homozygous mutant genotypes were selected by allele-specific PCR on F_2_ populations. The presence of the transgene was selected by histochemical assay for the expression of GUS activity (Jefferson *et al.*, 1987).

### Plasmid design and transformation

For analysis of *de novo RPS* methylation in *rdr2*, pGreen 0049 (Hellens *et al.*, 2000) harbouring a 35S–Luc reporter gene and a kanamycin resistance marker was used as a vector, digested with *Hin*dIII and ligated with a 1.6-kb RPS *Hin*dIII fragment isolated from p35S *GUS/RPS* (Muller *et al.*, 2002). Transgenic lines were isolated after transforming *rdr2* with the resulting construct pGreen49+RPS (Clough and Bent, 1998).

### Bisulfite sequencing

The sequence of the RPS region analysed by bisulfite sequencing is shown in Figure S2. Genomic DNA was isolated using a GenElute plant genomic DNA miniprep kit (Sigma-Aldrich, http://www.sigmaaldrich.com/) and subjected to bisulfite treatment using an Epitect bisulfite kit (Qiagen, http://www.qiagen.com/) according to the manufacturer’s instructions, except that the procedure was repeated twice to disrupt secondary structures and ensure complete conversion. A 1-μg aliquot of input DNA was used for the conversion reaction. In order to test whether this treatment leads to complete C→T conversion, 20 pg of an *RPS*-containing plasmid was mixed with 1 μg of wild-type DNA for a reconstitution control experiment, and complete conversion was confirmed.

To analyse the *RPS* top strand, primers *RPS*-top-F and *RPS*-top-R were used (Appendix S1). PCR was carried out using Go *Taq* polymerase (Promega, http://www.promega.com/) under the following conditions: 94°C for 4 min, 51°C for 2 min and 72°C for 1 min (two cycles), then 94°C for 1 min, 51°C for 2 min and 72°C for 1 min (38 cycles), generating a 421-bp product. PCR products were separated on a 1% agarose gel and the DNA was excised and cleaned up using a QIAquick gel extraction kit (Qiagen). The purified fragment was then cloned using a TOPO-TA cloning kit (Invitrogen, http://www.invitrogen.com/) according to the manufacturer’s recommendations, and recombinant plasmids were transferred into one shot MACH-Ti competent cells (Invitrogen). Transformants were selected on LB culture plates with 50 μg ml^−1^ kanamycin and 40 mg ml^−1^ X-gal, and colonies were selected for plasmid isolation using a QIAprep spin miniprep kit (Qiagen).

### Analysis of bisulfite-treated genomic sequencing lines

For each line, 9–20 clones were sequenced and sequences were exported into the BioEdit program (Hall, 1999). Aligned sequences were saved in FASTA format and were analysed by the MethTools2 program (http://methdb.igh.cnrs.fr/methtools/MethTools2_submit.html). The tab files returned by MethTools were pasted into an Excel spreadsheet to calculate and illustrate DNA methylation frequencies at individual cytosine residues. Bisulfite sequencing data were also analysed by the CyMATE programme (Hetzl *et al.*, 2007) and are presented in Figure S3.

### Analysis of methylation patterns in regions cloned after Gla I digestion of ddm1 DNA

The following regions were selected for bisulfite sequencing: At1g02010 – chromosome 1, positions 350 334–350 576; At3g53580 – chromosome 3, positions 19 877 246–19 877 428; At4g10140 – chromosome 4, positions 6 324 044–6 324 271; At4g14365, chromosome 4, positions 8 271 333–8 271 568. Sequence data are provided in Figure S2.

### Detection of small RNAs

About 50 μg of small low-molecular-weight RNA was isolated from rosette leaves (Hamilton and Baulcombe, 1999), separated on a 15% denaturing polyacrylamide gel, and transferred onto a Hybond Nx membrane (Amersham, http://www5.amershambiosciences.com/) by carbodiimide-mediated cross-linking (Pall *et al.*, 2007). A RNA single-strand probe was generated by T7 RNA polymerase transcription of a plasmid template in the presence of α^32^P-labelled UTP. Primers BsF (5′-CCCAACACCTTGGAATGATTGC-3′) and BsR (5′-AGGAGGTATCTGTCTTCTTTTTTAC-3′) were used for amplification of an RPS fragment, which was cloned into pGEM-T Easy vector (Promega). As a positive control, an oligonucleotide with sequence complementary to miR159 was labelled with T4 polynucleotide kinase and γ^32^P-ATP.

## Supporting information

**Figure S1.** Identification of mutant lines and list of oligonucleotides.

**Figure S2.** Sequence of regions analysed by genomic sequencing.

**Figure S3.** RPS bisulfite data in CyMATE format.
