# Supplementary material for: Cooperative activity of DNA methyltransferases for maintenance of symmetrical and non-symmetrical cytosine methylation in Arabidopsis thaliana
Source: Plant J. 2008 Aug 26;56(5):814–23. doi: 10.1111/j.1365-313X.2008.03640.x (PMC2667643; doi:10.1111/j.1365-313X.2008.03640.x)
Supplement: Supplementary file 1 [file tpj0056-0814-SD1.pdf]

Supplementary Data

Identification of mutant lines

ago4-1 (At2g27040) in *Landsberg erecta* ecotype background, EMS generated mutant with a C to T transition at 3774bp position. Genotyping was done by PCR using primers Ago4-F and Ago4-R followed by digestion with *Ava*II. Homozygous lines are phenotypically distinguishable due to lack of trichomes. For a homozygous line, a 1054bp line will be generated that will not be digested, while in wildtype the fragment will be digested generating a 607bp and a 447bp fragment.

cmt3-7 (At1g69770) in *Landsberg erecta* ecotype background, EMS generated mutant with a C to T transition at 800bp position. Genotyping was done by PCR using primers Cmt3-F and Cmt3-R, followed by digestion with *Cac*8I. For a homozygous line, a 744bp line will be generated that will not be digested, while in wildtype the fragment will be digested generating a 520bp and a 224bp fragment.

dcl3 (At3g43920) in *Columbia* ecotype background, T-DNA insertion line obtained from GABI-KAT (GABI_327D02). Genotyping was done by PCR using primers Dcl3-F and Dcl3-R. No product is obtained in a homozygous line but a 1kb fragment is obtained in a heterozygous or wild type line. The presence of the T-DNA was confirmed by PCR using primers Dcl3-R and GABI-KAT LB generating a 650bp fragment.

drd1-6 (At2g16390) in *Columbia* ecotype background was a kind gift from Dr. Matzke, EMS generated mutant with a G to A transition. Genotyping was done by PCR using primers Drd1-6 F and Drd1-6 R followed by digestion with *Nde*II. For a homozygous line, a 168bp product will be digested generating a 95bp and a 73bp fragment.

kyp2-1/SUVH4 (At5g13960) in *Landsberg erecta* ecotype background, EMS generated mutant with a G to A transition at position 4419 and G to T transversion at position at position 4422 creating a *Bgl*II site. Genotyping was done by PCR using primers Kyp2-F and Kyp2-R followed by digestion with *Bgl*II. Homozygous lines are phenotypically distinguishable due to a lack of trichomes. For a homozygous line, a 307bp product will be digested generating a 207bp and a 37bp fragment.

rdr2-2 (At4g11130) in *Columbia* ecotype background, T-DNA insertion line from SALK with an Accession number SALK_059661. Genotyping was done by PCR using primers Rdr2-F and Rdr2-R. For a homozygous line, no PCR product is generated, while a 899bp fragment is obtained in a heterozygous and wild type line. The presence of T-DNA was confirmed by PCR using primers Rdr2-F and SALK-LB generating a 556bp fragment.

ddm2-1/met1-1(At5g49160) in *Columbia* ecotype background was a kind gift from Dr. Mittelsten Scheid. The *met1-1* mutant was generated by EMS mutagenesis with a C to T transition (corresponds to position 3898 in AB016872). Genotyping was done by PCR using primers DDM2-1 F and DDM2-1 R followed by *Hae*III digestion. The met1-1 mutation destroys a *Hae*III site in the amplified fragment to yield a 121bp and a 298bp fragment for a homozygous line, while for a wildtype line a 80bp, a 121bp and a 218bp fragment is generated.

drm1 (At5g15380) *drm2* (At5g15380) in *Wassilewskija* ecotype background is a double mutant kindly provided by Dr. Mittelsten Scheid. Genotyping for DRM1 was done by PCR using primers Drm1-F and Drm1-R. For a homozygous line, no PCR product is obtained while a 650bp fragment is obtained in a heterozygous or wild type line. The presence of T-DNA was confirmed by PCR using primers Drm1-R and TL2 generating a 750bp fragment. Genotyping for DRM2 was done by PCR using primers Drm2-F and Drm2-R. For a homozygous line, no PCR product is obtained while a 750bp fragment is

obtained in a heterozygous or wild type line. The presence of T-DNA was confirmed by PCR using primers Drm1-R and TL2 generating a 650bp fragment.

**Oligonucleotides**	In 5' to 3' orientation
**Genotyping**	
Ago4-F	TGACTGACAGCTGAAAATGGGATGTGGAT
Ago4-R	GCCACTCCCTAGAACTCACCA CCTAAGTT
Cmt3-F	TTGACTACCCCGGGAATGAACCCATTTGT
Cmt3-R	GATCTGCAACAAATCTCAGC
Dcl3-F	ATGCATTCGTCGTTGGAGCCGGAG
Dcl3-R	TGCACTTCTAAGGACATCCAATAA
GABI-KAT LB	CCCATTTGGACGTGAATGTAGACAC
Drd1-6 F	AGCTAAGGGATGGAAACT AGG
Drd1-6 R	CGAGATGCTCCAACAAGCGCG
Kyp2-F	GCAGTGAAG ATGAGAATGCGCCAGAGTTC
Kyp2-R	CGCTATCAAGCGCATATCCATAGTCGTAAGTGAGATC
Rdr2-F	CTGCATCAGCTTATAAAAGATTG
Rdr2-R	CTTCAAGCATAT TCCCAAGCATAG
SALK-LB	AACCAGCGTGGACCGCTTCTG
DDM2-1 F	CTCTTTAGTAGAAGTTGGCATG
DDM2-1 R	ATATGTATGTATAGATATTTTCTCC
Drm1-F	CGT GAA TCA GAG GAC ATG AAC
Drm1-R	AAC CGG AGC CGT CTC ATC AA
Drm2-F	GGT GTA GGC CTT AGA TGT GT
Drm2-R	GGTAGACGAATCGGCTCGTCATC
TL2	TGGACGTGAATGTAGACACGTCG
**Bisulfite Sequencing**	
RPS-top-F	CTg/aTATTTTTCTCCCTTCA
RPS-top-R	AAGTAGAAAAGGAAAGAGAAAAGGGG
RPS-low-F	ATATACATACATATATATACACT
RPS-low-R	AGTTTAGTGGTTATTATTT
