# Supplementary material for: Cooperative activity of DNA methyltransferases for maintenance of symmetrical and non-symmetrical cytosine methylation in Arabidopsis thaliana
Source: Plant J. 2008 Aug 26;56(5):814–23. doi: 10.1111/j.1365-313X.2008.03640.x (PMC2667643; doi:10.1111/j.1365-313X.2008.03640.x)
Supplement: Supplementary file 2 [file tpj0056-0814-SD2.pdf]

RPS
CCCAACACCTTGGAATGATTGCTGGCAGAACATGGCAGAACAGCAGAACAAAATGTTAC
GTAGCTTGTGAGATTCGAACCCACAACATTGAGGTTGATGTTTGAGCCAGCAACCAGTG
GCACCATGAGCCATTTTGTTTAGTAGGTGCCACTTTAAAATATAGATTGAAATACACTG
TCAAGACTGATATACATACATATATATACACTGTCGCGATTGAGGGTAGCGCGTACCAT
GGCACCCCCTACCCTCAATGTAGATCCGCCCCTGGATACCTCTGAGCTGTACAAAAGAA
ATTACAGCTGACTACCCAACATACTAGGTTTAGAGTTATCCAGACTAGACATCTAAGAG
TAAAAAAGAAGACAGATACCTCCT

At1g02010
ACAAACCTCTACATGAGTGGAGAGTTTGTCAACTTGTTCTCCATATTGCGGCAAAGCCT
GAACTATTTTCTGTAGATCTCGTGTAGACAACTCGCTACCGTCCCTGCGTCACATTAGC
CAAAGATGTTAGCAATACATATCAAAAAAGTCTGTGAGGGATCACGAATCCATATCGAG
GATAACAAGAAACTTAAAGTCTTCCAGATATCAAACTTAGGCAACTGTTCCATTGTCTA
AGCACTC

AT3g53580
CGCGGAGCAGGTCCGCAAATTCCCCCCAATCTTCGAATATAATTCTCAGTTGATTATAA
ACAGTCTCTGTATCCTTATATGCTTAATGCTTACGCATACTTAAAACTGCAGGAGCAAC
TTTGGCCTGTGGAACTGGAGCTTGTGCTCTGGTTGTTGCAGCAGTCCTTGAAGGTCGAG
CCGACA

AT4g10140
CTACGAGCCACCCTAGTAACTGCAATAACCATAGGACTTAAAATAAGACTTGCAAGTTA
CTAATGATAAGAGCACAAAAGATTAGGCTAGGGAATGCAAAAAGCGGTTCGACATAGTT
CCCCCATTAGCTAAGAGAAACGAGACTCATTGTAGCTACTTACCGCATTACCAATGCTT
AAAAAGACTTCTTGAGAGGCTTGACCAGTGAAAACCTTCTTTAGAGCATCT

AT4G14365
GTCTTAGATTCGAAAAACTCCAAACATACTAACAAAATGTATAATGGGTCAGAAGGGCT
TATAGGATCGATGCGCTGTAACGCCAACTGAATTCCACGGAACAGGTTGTTACATATAG
AGTTTGTAAGATTGAAGGAAAAATTAACATTGATCATGTAACAAAGATAAAAAGACAAA
CTAAACTTGTCGTGATTCTATATTTCGACTTCTTTGACTTCACCACATAACATACACAC
