# Supplementary figures and images for: Cooperative activity of DNA methyltransferases for maintenance of symmetrical and non-symmetrical cytosine methylation in Arabidopsis thaliana
Source: Plant J. 2008 Aug 26;56(5):814–23. doi: 10.1111/j.1365-313X.2008.03640.x (PMC2667643; doi:10.1111/j.1365-313X.2008.03640.x)

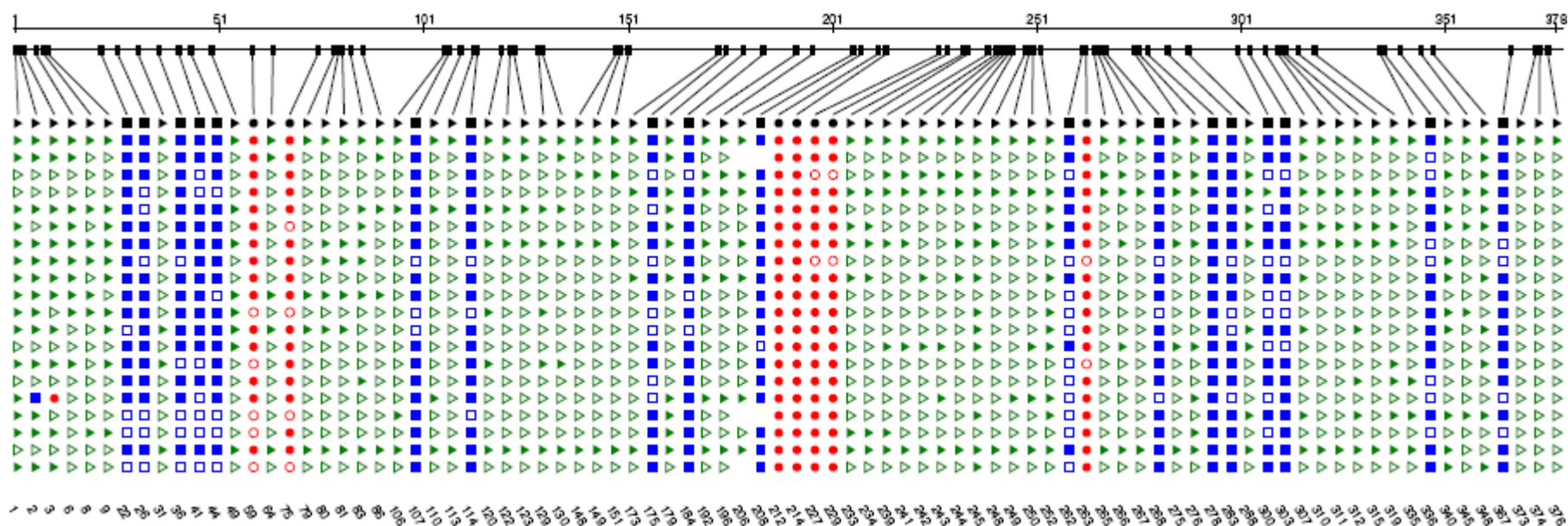

WILDTYPE, 20 clones

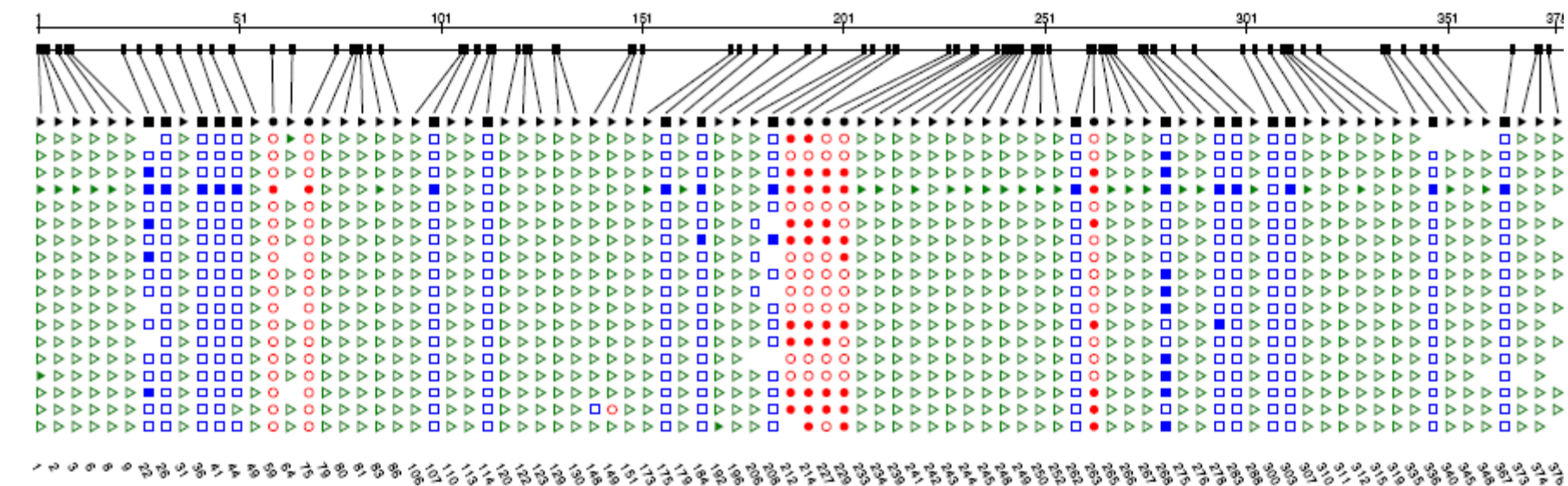

*drd1*, 18 clones

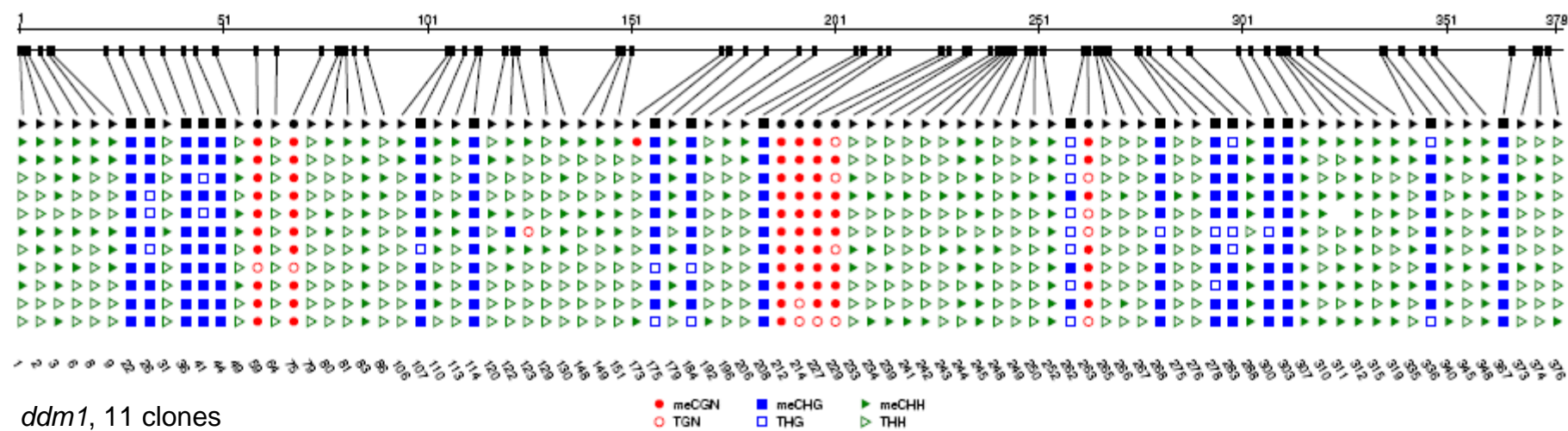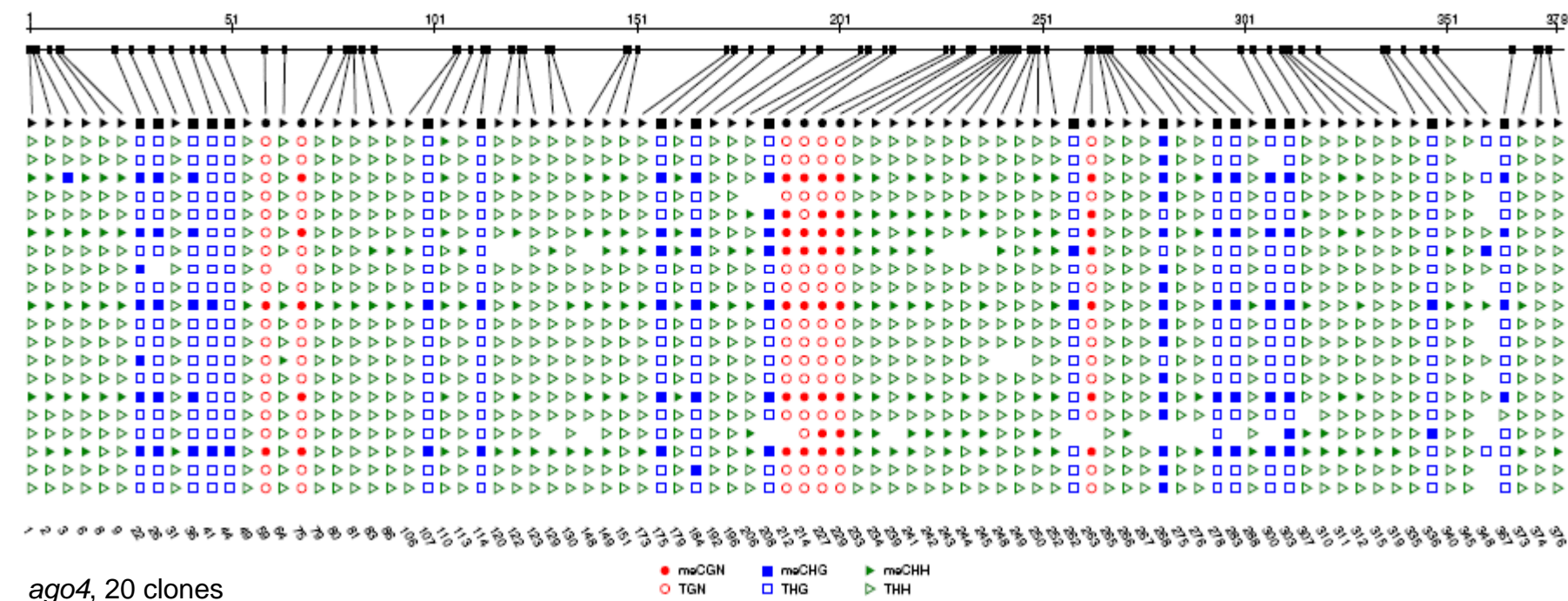

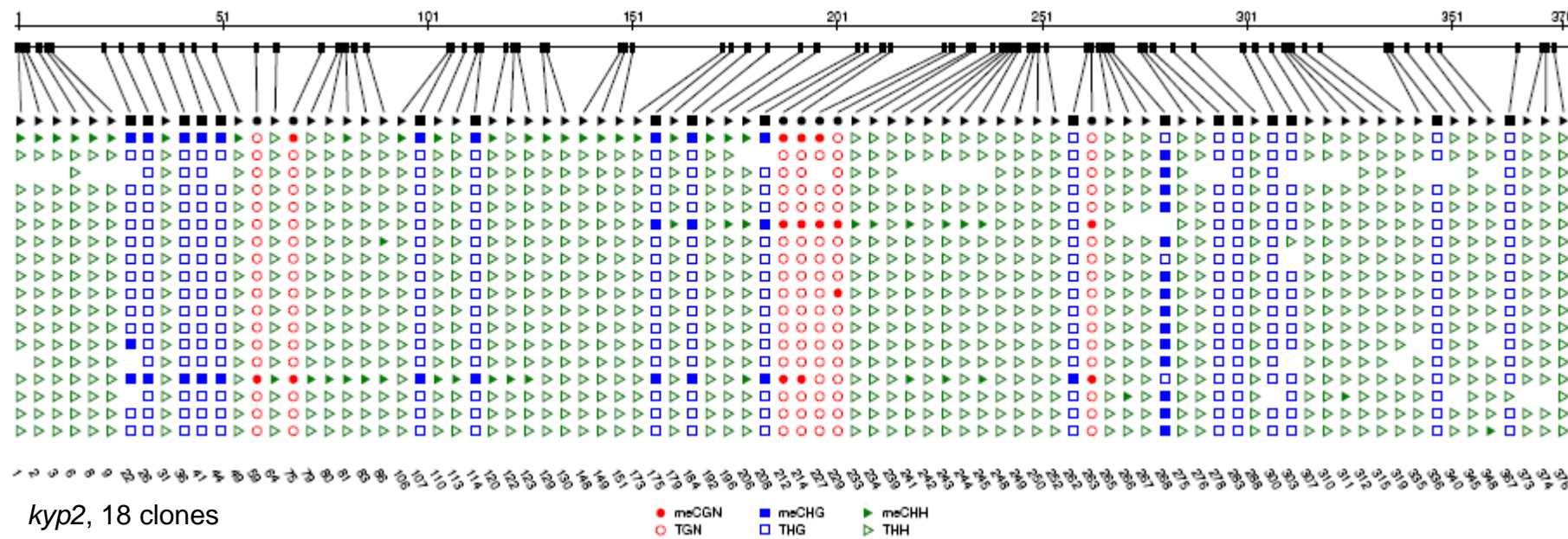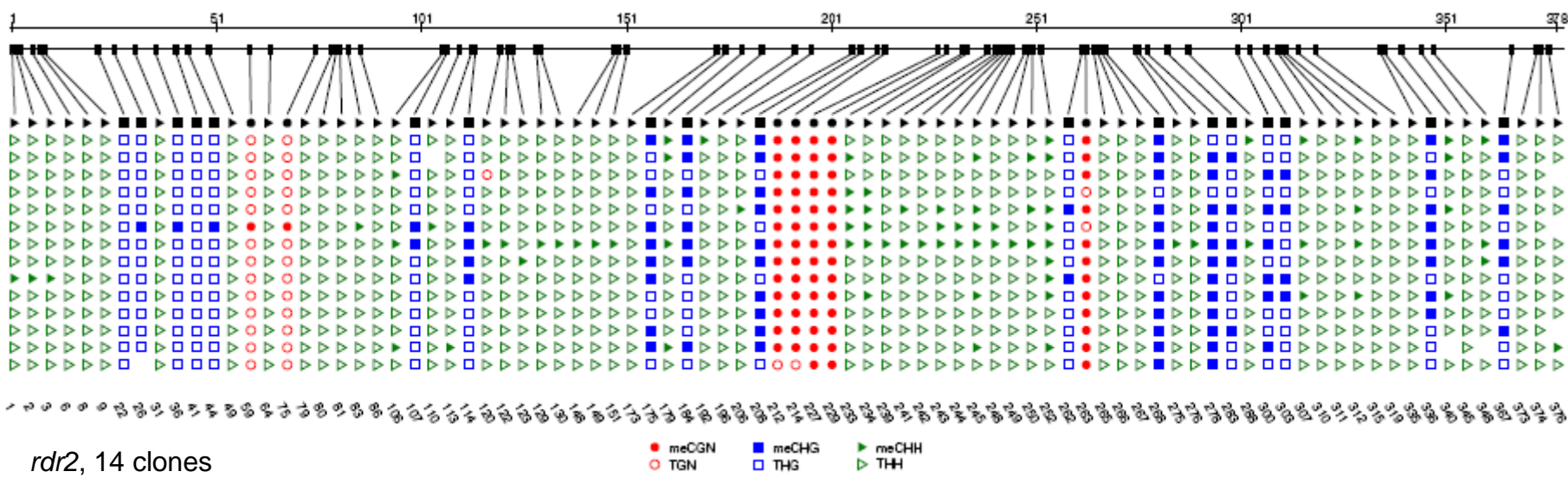

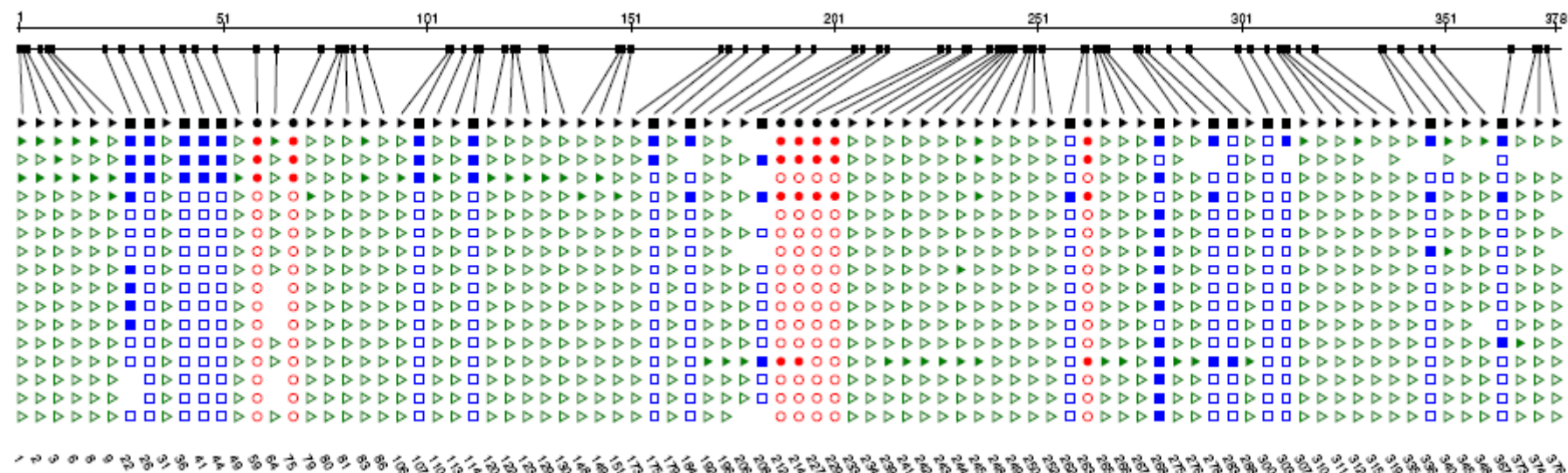

*dc13*, 16 clones

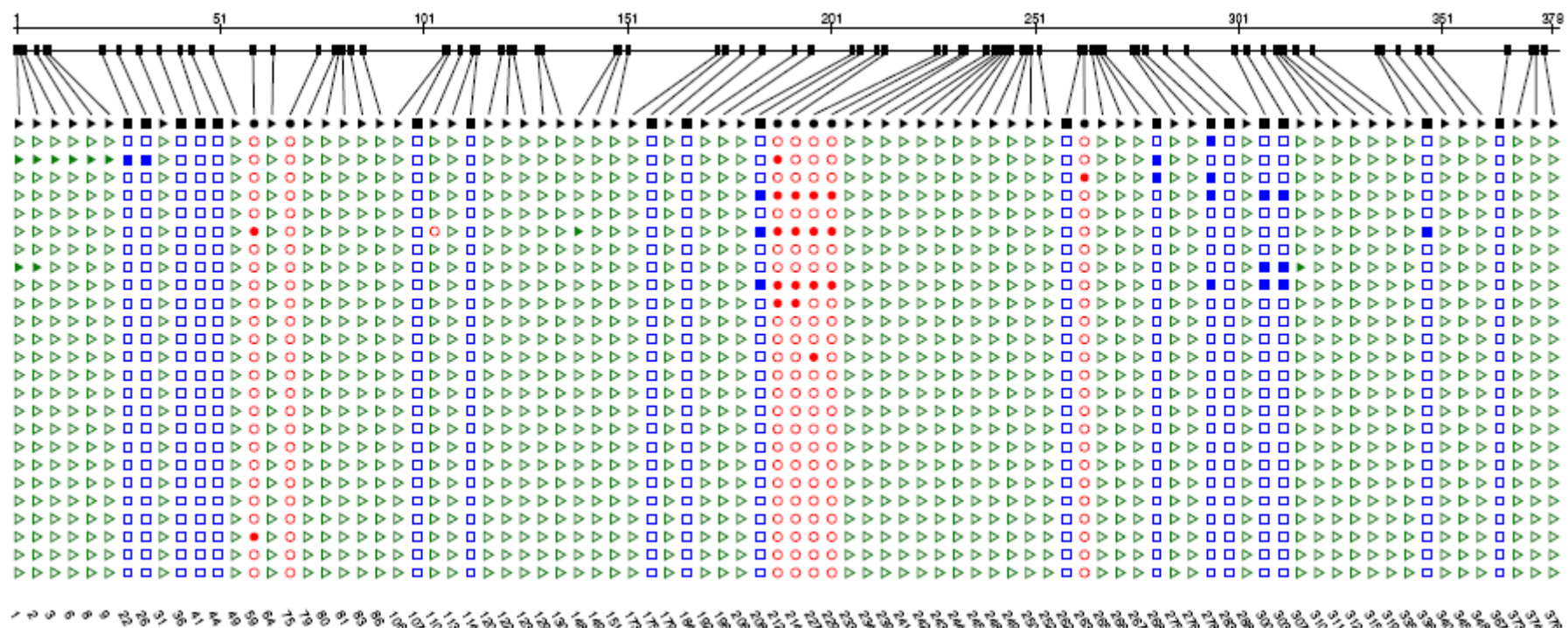

*met1*, 25 clones

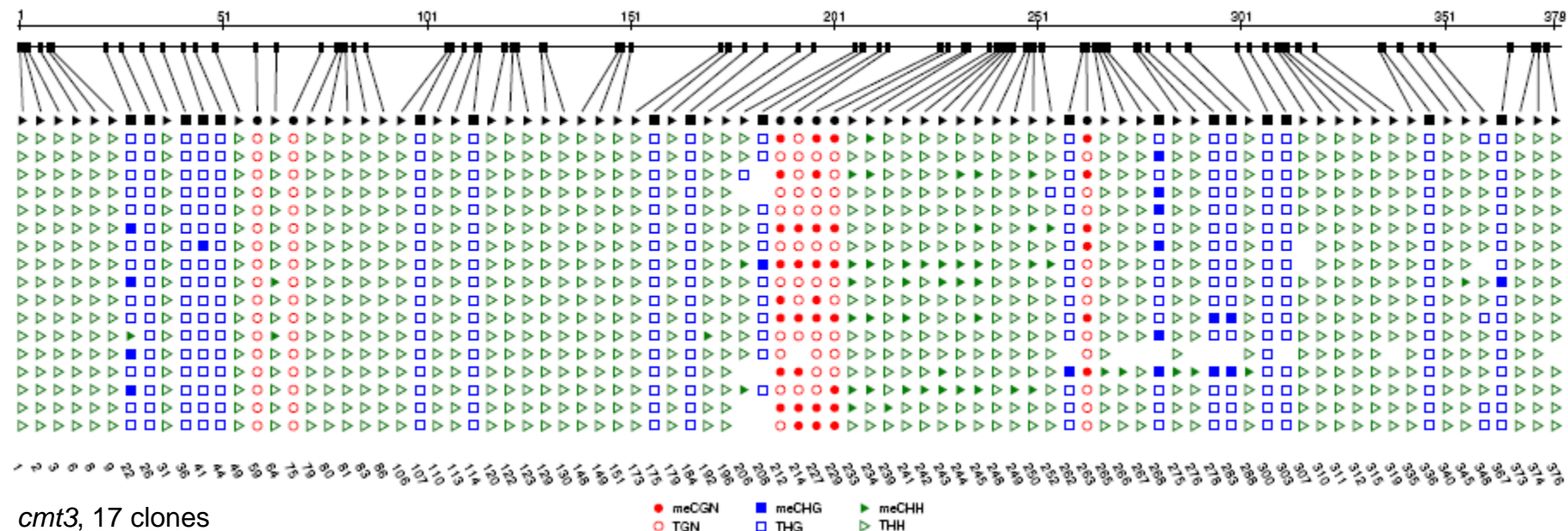

*cmt3*, 17 clones

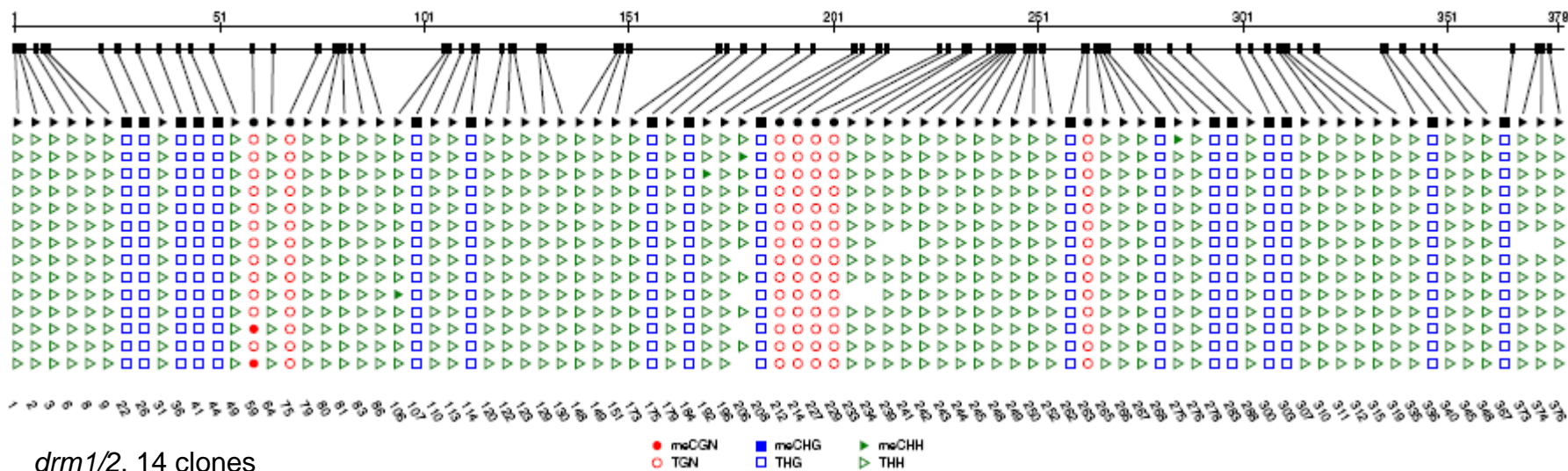

*drm1/2*, 14 clones

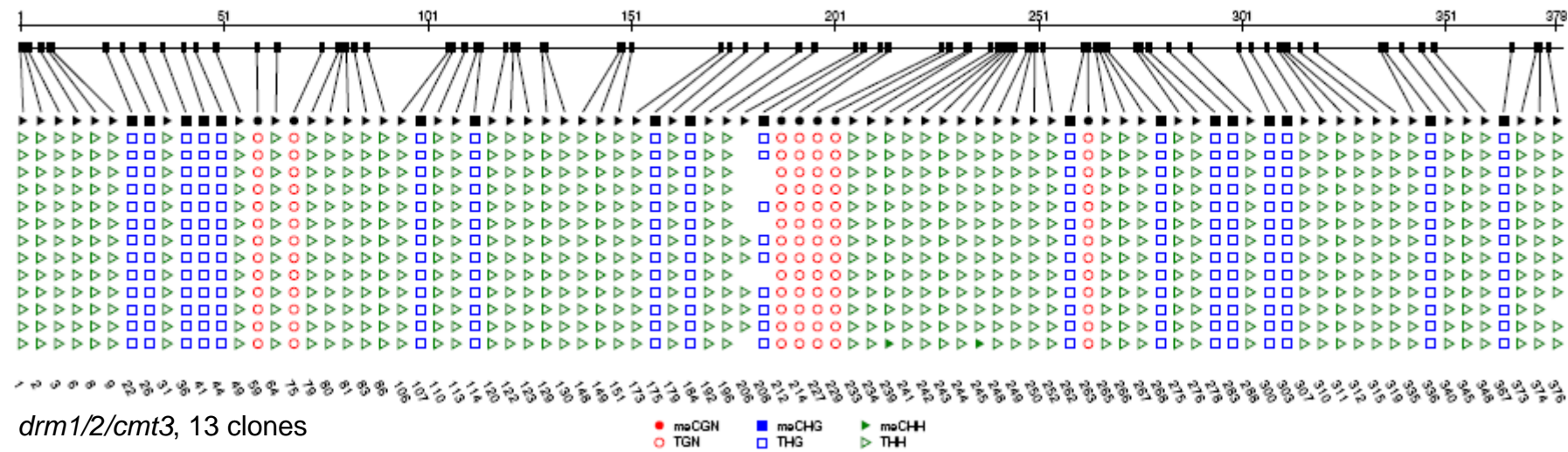

Supplement: Supplementary file 3 [file tpj0056-0814-SD3.pdf]
